# Supplementary material for: Pre-pandemic geographic access to hospital-based telehealth for cancer care in the United States
Source: PLoS One. 2023 Jan 31;18(1):e0281071. doi: 10.1371/journal.pone.0281071 (PMC9888704; doi:10.1371/journal.pone.0281071)
Supplement: Attachment — Submitted filename: Geographic Access Telemedicine Revisions Letter PLoS ONE.docx [file pone.0281071.s001.docx]

Dear Dr. Bhaskar,

Thank you for allowing us to revise this manuscript, “Geographic Access to Hospital-Based Telehealth for Cancer Care” for consideration at *PLoS ONE*.

We believe that we have been able to address all reviewers’ and editors’ comments; please see below for point-by-point responses. Please note that the funders of this work had no role in study design, data collection and analysis, decision to publish, or preparation of the manuscript. Funding and sources of competing interests do not alter our adherence to all PLOS ONE policies on sharing data and materials.

All page/line numbers refer to the version of the manuscript with changes tracked.

Thank you again for your consideration. Please contact me if any questions arise or if further clarification is needed.

Sincerely yours,

David Shalowitz, MD, MSHP

[david.shalowitz@gmail.com](mailto:david.shalowitz@gmail.com)

**RESPONSES TO REVIEWERS:**

**Editorial review:**

1. Please ensure that your manuscript meets PLOS ONE's style requirements, including those for file naming.

*Done.*

2. Please ensure that you have specified (1) whether consent was informed and (2) what type you obtained (for instance, written or verbal, and if verbal, how it was documented and witnessed). If your study included minors, state whether you obtained consent from parents or guardians. If the need for consent was waived by the ethics committee, please include this information.

*Done. This study does not qualify as human subjects research (only hospital data are involved), and thus did not require ethics committee/IRB review (lines 142-143).*

3. Please amend your current ethics statement to include the full name of the ethics committee/institutional review board(s) that approved your specific study.

*See answer to #2, above.*

4. Please state what role the funders took in the study.  If the funders had no role, please state: "The funders had no role in study design, data collection and analysis, decision to publish, or preparation of the manuscript."
   If this statement is not correct you must amend it as needed. 
   Please include this amended Role of Funder statement in your cover letter; we will change the online submission form on your behalf.

*Done. The above statement has been included above in our revisions/cover letter.*

5. Please confirm that this does not alter your adherence to all PLOS ONE policies on sharing data and materials, by including the following statement: "This does not alter our adherence to  PLOS ONE policies on sharing data and materials.” (as detailed online in our guide for authors <http://journals.plos.org/plosone/s/competing-interests>).  If there are restrictions on sharing of data and/or materials, please state these. Please note that we cannot proceed with consideration of your article until this information has been declared.

*Done. The above statement has been included in our revisions/cover letter above.*

6. Please include your updated Competing Interests statement in your cover letter; we will change the online submission form on your behalf.

*Done. See above.*

7. We note that you have stated that you will provide repository information for your data at acceptance. Should your manuscript be accepted for publication, we will hold it until you provide the relevant accession numbers or DOIs necessary to access your data. If you wish to make changes to your Data Availability statement, please describe these changes in your cover letter and we will update your Data Availability statement to reflect the information you provide.

*We would like to amend our data availability statement to provide raw data or sources to obtain raw data (e.g., the American Hospital Association data set) on request by contacting the corresponding author.*

8. Please include a caption for figure 1.

*Figure 1 should be captioned “County-level availability of hospital-based telehealth and oncology service in the United States.”*

9. We note that Figure 1 in your submission contain [map/satellite] images which may be copyrighted.

*Figure 1 is entirely original data produced for this manuscript and is not copyrighted.*

10. Please include your tables as part of your main manuscript and remove the individual files. Please note that supplementary tables (should remain/ be uploaded) as separate "supporting information" files

*Done.*

**Reviewer #1**

1. It is not clear if the availability of telehealth services correlate with the quality of the care provided at a given hospital. Could the authors comment on the quality of the services provided by these hospitals?

*Thank you for this comment. Unfortunately, while this correlation is important, outcomes are not included in this dataset. We were therefore only able to comment on telehealth availability per se as a potential indicator of quality cancer care.*

2. Do the hospitals that provide telehealth services, use telehealth to provide oncology care? Are there data to show what the telehealth services in these hospitals were used for in the pre-pandemic era?

*Thank you for this comment. See lines 212-217: The AHA data do not specify whether telehealth was used specifically for cancer care (or any specific disease processes other than addiction services). We therefore limit our conclusions to whether hospitals had, during the study period, the *capacity* to utilize telehealth for cancer care. Contemporary data for specific use-cases of telehealth are very limited. We were only able to find nationwide data regarding proportion of telehealth claims for oncology in 2021 (8%, see lines 231-232). We advocate for inclusion of these data points in future versions of the AHA survey to address this issue (lines 216-218).*

3. It is difficult to assess policy/clinical implications of this work without knowing how the lack of availability of services/care relate to cancer outcomes--how do the differences in availability of services translate to disparities in cancer outcomes?

*Thank you for this comment. Unfortunately, as above, clinical outcomes were not available within the AHA dataset; we are therefore unable to directly show correlation between hospital-based telehealth services and cancer outcomes. However, geographic barriers to care (one of the primary drivers of the utility of telehealth) are robustly associated with worsened cancer outcomes (lines 68-72) and telehealth has been identified as a critical component of improving rural cancer care (lines 75-78).*

4. The results show that larger hospitals are 37 times more likely to provide oncology services among those hospitals that provide telehealth. Most of the smaller/rural hospitals are less likely to provide oncology care or access to telehealth services—perhaps these hospitals are more likely to refer cancer patients to nearby larger hospitals for cancer care? If that is true, the availability of telehealth services in larger hospitals that provide oncology services could perhaps help reduce cancer care deficiencies.
   Overall, it is not clear if the greatest improvement in health outcomes depend on increasing oncology and telehealth services in rural hospitals OR further increasing the telehealth capacity in existing larger hospitals. It is not clear how a policy-maker could use this study to improve care for the population.

*Thank you for this comment. We agree that there could be various models of telehealth cancer care, including outreach from referral centers, outreach from community/rural hospitals, and clinician-to-clinician telemedicine between hospitals. Referral for in-person care (as mentioned by the reviewer) at referral centers is also certainly possible, but is associated with burdens for the patient and associated with worsened cancer outcomes (via geographic barriers).*

*We recommend next steps for cancer care researchers and policymakers in lines 187-205 and 216-218.*

**Reviewer #2**

1. The title does not contain the place of the study.

*We have amended the title to “Geographic Access to Hospital-Based Telehealth for Cancer Care in the United States”*

2. The authors did not mention study design which is a cross-sectional.

*We now specify “cross-sectional” in the Abstract and on line 103.*

3. There are several errors in the tables’ design and data presentation.

*We have reviewed these tables and are unable to find the errors mentioned by the reviewer. We would be happy to address anything specific identified in the copy editing process.*

4. The reference list is incomplete.

*We are unfortunately unable to address this concern without specifics.*

**Reviewer #3**

1. Thank you for acknowledging that these data are pre-pandemic and therefore likely do not represent the current use of telemedicine in hospitals. This is a significant limitation given what we know about the uptake of telemedicine in the US since 2020, and should be reflected in the title. Further, in order to make this relevant to today’s researchers and policy makers, the authors should consider bringing this limitation to the forefront of the discussion-- currently it is not mentioned until late in the discussion. For example, in the first discussion paragraph, when you discuss increasing rurality being associated with lower telehealth rates, I suggest you discuss how this disparity is likely still the case today (some data should be available to support this), even though your analysis is pre-COVID

*Thank you for this. We have added “Pre-Pandemic” to the manuscript title and line 81, and “prior to the SARS-CoV-2 pandemic” to the Objective in the abstract. We also added “Moreover, few data exist on the availability of hospital-based telehealth services prior to their rapid adoption during the SARS-CoV-2 pandemic beginning in 2020” to the Introduction, lines 80-82.*

*We have also added to line 185 “Although data are lacking on sustained, post-pandemic implementation of telehealth for cancer care, it is likely that access disparities continue for rural populations.*

2. In the second discussion paragraph, the authors write about how it is “crucial to encourage hospitals caring for cancer patients” to adopt telehealth. Again, this needs to be framed in the understanding that this may have already happened. Further, there should be more specifics included. For example, what do the authors mean by “encourage?” Through national policy? Medicare reform? There is a robust discussion currently around making certain telemedicine reimbursement waivers permanent beyond the Public Health Emergency, and this might be a good place to bring in some of these arguments to describe how hospitals would be encouraged to adopt these technologies.

*Thank you for this suggestion. Lines 187-188 now read “It is crucial to encourage hospitals caring for cancer patients without the availability of telehealth to sustainably adopt this critical adjunct to cancer care, if they have not already done so post-pandemic.”*

*Lines 203-205 now read “The loosening of Medicare telehealth regulations and promotion of payment parity during the SARS-CoV-2 pandemic, the potential for those regulations to be extended beyond the pandemic, the flow of pandemic-related telehealth funding, and teleoncology innovations may help incentivize expansion of telehealth services in the future.”*

3. The authors point out that only 8% of telehealth claims were for oncology in 2021. This statement needs to be contextualized in comparison to other specialties, and regarding what percent is appropriate for oncology. For example, my understanding is that an initial visit for a cancer diagnosis needs to be done with an in person exam. Are there initial visits that are appropriate for telemedicine? Further, rural patients who are on active treatment typically have their visits stacked on the same day as infusion, radiation, or radiology visits (see: DeGuzman et al, (2015). Identifying barriers to navigation needs of cancer survivors in rural areas. Journal of Oncology Navigation & Survivorship, 6, 34-42), so a telehealth visit may not make sense in these cases.

*Thank you for this comment. We respectfully contend that there is no objectively appropriate proportion of all telehealth claims allocated to oncology services, though this would be an interesting area for further study. With regard to which visits may be appropriate candidates for telehealth, our previous work suggests that any non-procedural visit might be accomplished virtually, but this is (as the reviewer suggests) an area of some controversy and somewhat outside the scope of this manuscript which focuses on hospital-based telehealth and oncology services.*

4. It may be worthwhile to consider discussing that even as telehealth becomes more ubiquitous in hospitals, those living in rural areas often lack broadband, which raises its own set of challenges. Since COVID-19, alternative origination sites (such as public libraries), and the need to extend federal regulations that support reimbursement for telemedicine services.

*Agreed. Lines 205-206 now read “Importantly, telehealth expansion should occur in the context of broader efforts to improve digital health equity, including increasing availability of broadband internet access for rural populations.” We have added mention of parity in reimbursement in our response to comment #2, above.*

5. In the section on Data Sources (page 4), it would be clearer to list the data sources in the same order that you explain them in that section (i.e. switch #2 and #3)

*Done.*

6. On page 5, line 133, it would be clearer to state what the outcomes are before explaining you they were derived: “Thus study has two primary outcomes: x and y.”

*Done.*

7. On page 5 lines 140-141, it would be clearer to put the statement “per their self-reported telehealth and oncology services provisions” in parenthesis

*Done.*

8. On page 8, line 199, although I was able to figure out where you got the number 2326 from in your table (hospitals providing cancer care), it took a while to figure it out and I wonder if this could be explained clearer.

*Done. Revision reads “2,054 out of 2,326 (88.3%) of hospitals providing cancer care also provided care by telehealth, whereas 272 hospitals offered cancer care but no telehealth.”*
